# Barriers and facilitators to viral load suppression among adolescents and young people living with HIV receiving intensive adherence counselling in east central Uganda: A COM-B model-informed qualitative study

**DOI:** 10.1371/journal.pgph.0007131

**Published:** 2026-08-20

**Authors:** David Livingstone Ejalu, Nangendo Joan, Peter Simon Okello, Sabrina Bakeera-Kitaka, Achilles Katamba, Joan Kalyango, Adithya Cattamanchi, Fred C. Semitata, Moses R. Kamya, Anne R. Katahoire

**Affiliations:** 1 Department of Clinical Epidemiology and Biostatistics, School of Medicine, College of Health Sciences, Makerere University, Kampala, Uganda; 2 Department of Epidemiology and Biostatistics, School of Public Health, College of Health Sciences, Makerere University, Kampala, Uganda; 3 Department of Medicine, University of California Irvine, Irvine, California, United States of America; University of California Irvine, UNITED STATES OF AMERICA

## Abstract

Suboptimal viral load (VL) suppression among adolescents and young people living with HIV (AYPLHIV) remains a major challenge to HIV control in Uganda. Although Intensive Adherence Counselling (IAC) is implemented as a structured intervention for individuals with unsuppressed viral loads, suppression rates among AYPLHIV remain low. Evidence on the contextual factors shaping VL suppression after IAC remains limited. This study explored barriers and facilitators to optimal adherence and engagement in IAC as a pathway to VL suppression among previously non-suppressed AYPLHIV aged 10–24 years who had undergone IAC in East-Central Uganda. A descriptive exploratory qualitative study design was conducted among participants purposively sampled across four districts. Data collection and analysis of individual-level factors were guided by the Capability, Opportunity, Motivation - Behaviour framework, while health system factors emerged inductively and complemented the COM-B framework. In total, 8 AYPLHIV, 4 caregivers, and 10 health workers participated. Interviews were audio-recorded, transcribed verbatim, translated into English, and analyzed using thematic analysis. Most participants (64%) were female, and the median age of AYPLHIV was 16.5 years (interquartile range [IQR]: 14.5-20.5). Multiple individual-level factors influenced VL suppression. Capability barriers included forgetfulness, while facilitators included reminder routines. Opportunity barriers comprised economic hardship, parental resentment and social class-based reluctance, and non-disclosure of HIV status, whereas caregiver support was a facilitator. Motivation barriers included emotional distress and substance abuse, while facilitators included future aspirations and religious beliefs. Health system barriers included a rigid IAC delivery schedule, and long waiting time, whereas peer support and WhatsApp groups acted as facilitators. The findings highlight the need to redesign IAC from a predominantly facility-based intervention to an adolescent-responsive model that integrates peer-led engagement and flexible community-based service delivery to optimize viral suppression among high-risk AYPLHIV. Key words: viral load, intensive adherence counselling, suppression.

## Introduction

Adolescents and young people living with HIV (AYPLHIV) continue to bear a disproportionate burden of the global HIV epidemic. An estimated 4.77 million individuals aged 10–24 years are living with HIV worldwide, with the vast majority residing in sub-Saharan Africa (SSA), which accounts for approximately 84% of AYPLHIV [1,2]. In Uganda, about 235,000 AYPLHIV are living with HIV, of whom 68% (160,000) are female [3]. Despite expanded access to antiretroviral therapy, viral load (VL) suppression among AYPLHIV remains suboptimal and poses a critical barrier to HIV epidemic control. Globally, only 47% of children living with HIV achieved VL suppression in 2024, compared to 74% of adults [4]. This gap is more pronounced in SSA, where approximately 43% of AYPLHIV are virally suppressed, relative to 76% among adults [1,4,5]. In Uganda, VL suppression among AYPLHIV remains below the UNAIDS 95-95-95 targets, with marked regional disparities, ranging from 48.8% in East Central Uganda to 70.0% in the North Eastern region [6,7].

The Uganda Ministry of Health is implementing Intensive Adherence Counselling (IAC) across antiretroviral therapy (ART) facilities to improve VL suppression among people living with HIV with unsuppressed VL. IAC is a structured, client-centred intervention designed to support individuals in identifying adherence barriers and co-developing tailored solutions to optimize ART adherence [8]. Delivery is predominantly facility-based, with follow-up sessions are conducted at monthly intervals, [9]. The counselling process is guided by the “Five A’s” framework (assess, advise, assist, agree, and arrange) which structures identification of adherence barriers, provision of targeted guidance, prioritization of feasible solutions, development of adherence plans, and scheduling of follow-up and ART refills [10,11].

Despite the scale-up of IAC, a large proportion of AYPLHIV remain virally unsuppressed following completion of the intervention. Evidence from Uganda across settings indicates low post-IAC VL suppression, with reported rates ranging from 13% in rural contexts to 66% in urban populations [12–14]. Although prior studies have identified broad barriers to ART adherence, the context-specific and mechanism-driven factors shaping IAC effectiveness in high-burden settings remain inadequately established [15,16]. This gap is particularly prominent in East Central Uganda, which consistently records the lowest national VL suppression rates [6,7]. Guided by the Capability, Opportunity, and Motivation-Behavior (COM-B model), this study examined individual and health system-level barriers and facilitators to ART adherence and IAC engagement as critical pathways to VL suppression among AYPLHIV. The findings aimed to inform development of contextually responsive strategies to optimize IAC delivery and improve virological outcomes in this high-burden setting.

## Materials and methods

### Ethics statement

Ethical approval for this study was obtained from the Makerere University School of Medicine Research and Ethics Committee (approval number Mak-SOMREC 2022–396) and the Uganda National Council for Science and Technology (approval number HS3807ES). Written informed consent was provided by all participants aged 18 years and older. For participants aged 10–17 years, consent was obtained from a parent or legal guardian, together with age-appropriate assent from the adolescents.

### Study design and theoretical framework

This study employed a descriptive qualitative design guided by the COM-B model [17]]. The primary target behavior was ART adherence during IAC, and the secondary target behavior was continuous engagement in IAC, both of which influence the achievement of IAC. The model was selected for its explicit conceptualization of adherence behavior as an interaction between three domains: Capability encompasses the psychological and physical capacity to adhere to ART, including knowledge and medication management skills. Opportunity refers to external factors, both physical and social, that facilitate or constrain adherence, such as health system structures and social support. Motivation comprises reflective and automatic processes, including beliefs, and emotions. Compared with models that primarily emphasize cognitive determinants, the COM-B framework was appropriate for this study as it offered a more comprehensive lens by integrating psychological, structural, and affective influences operating across individual and health system levels.

### Study setting and participants

The study was conducted in a resource-limited setting in east-central Uganda, encompassing Jinja District, Kamuli District, Mayuge District, and Jinja City. This area, commonly referred to as the Busoga sub-region, lies northeast of Kampala and is predominantly inhabited by the Basoga, who speak Lusoga. The region has consistently reported lower VL suppression rates relative to other regions in Uganda [6,7,14]. Participants comprised previously non-suppressed AYPLHIV aged 10–24 years who had undergone IAC, alongside their caregivers and health workers. Participants were recruited from health facilities across sub-county (Health Centre III), county (Health Centre IV), and district (hospital) levels.

### Sampling strategy and sample size

We employed purposive sampling with maximum variation to capture diverse perspectives. Rather than seeking proportional representation across participant groups, sampling maximized heterogeneity across facility level (Health Centre III, Health Centre IV, hospitals), geographic location (urban/rural; ≤ 5 km vs > 5 km from facilities), demographic characteristics (age, sex, school attendance), and stakeholder category (AYPLHIV, caregivers, healthcare providers) [18]. Eligible AYPLHIV included those who had achieved or not achieved VL suppression. Individuals who were clinically unstable or had advanced HIV disease were excluded. Caregivers comprised parents or guardians regardless of HIV status but excluded those not disclosed to by AYPLHIV. Healthcare providers included those directly involved in IAC delivery and had at least 6 months’ clinic experience, corresponding to at least one complete IAC cycle. Sample size was determined by data saturation, defined as the point at which additional interviews yielded no new themes or codes [19]. Data collection and analysis proceeded concurrently, with ongoing preliminary thematic analysis to assess saturation within each participant category. Interviews continued until saturation was reached, without a predefined sample size.

### Data collection procedures and quality assurance

Data were collected through face-to-face interviews at health facilities using COM-B informed guides to ensure systematic exploration of theoretically relevant factors. Interviews were conducted between 20^th^ September 2024–15^th^ January 2025 at health facilities. The interview approach was open and flexible to allow participants to introduce experiences and contextual influences that were not explicitly anticipated within the predefined framework. Guides for AYPLHIV and caregivers were translated into Lusoga using forward - backward translation by two independent translators to ensure conceptual equivalence [20]. Health worker interviews were conducted in English. All guides were pretested in two non-participating facilities in Jinja City with four participants, and their feedback informed final revisions. Eligible participants were contacted by phone and were provided with study information and scheduled for interviews at their treatment facilities. Interviews were audio-recorded, transcribed verbatim, and translated into English for analysis. Some of the participant quotations were edited to improve readability after translation. Transcripts were cross-checked against audio recordings to ensure accuracy and completeness. Methodological rigor was ensured using established qualitative strategies. Credibility was enhanced through member checking with three purposively selected AYPLHIV, one counsellor, and one nurse. Dependability and confirmability were strengthened through independent coding by the PI and an experienced qualitative researcher, with four consensus meetings to resolve discrepancies and document analytic decisions. Transferability was supported through maximum variation sampling across age, sex, ART duration, and care settings. Reflexivity was maintained through documentation of prior assumptions, a reflexive journal, and daily debriefing sessions to identify emerging insights.

### Data analysis

Data were analyzed using a thematic analysis approach, integrating both deductive and inductive coding strategies to identify patterns and meanings within the dataset. Deductive coding was guided by the COM-B model to capture individual-level factors, while inductive coding allowed for the identification of additional themes, particularly health system factors not fully encompassed within the COM-B framework. Data management and coding were facilitated using ATLAS.ti. An iterative analytic process was employed, conducted concurrently with data collection to allow for emerging insights from preliminary analyses to inform subsequent interviews. The analysis was guided by the six-phase framework developed by Virginia Braun and Victoria Clarke (2006) [21]. First, the research team engaged in data familiarization through repeated reading of transcripts to achieve immersion and identify preliminary patterns related to adherence and VL suppression. Second, two analysts, a senior public health researcher with expertise in qualitative methods and the principal investigator, independently coded seven purposively selected transcripts (approximately one-third of the dataset). Third, initial codes were compared and refined through collaborative discussion. Discrepancies were resolved through consensus, resulting in the development of a structured and well-defined coding framework. Fourth, codes were systematically organized into candidate themes by grouping conceptually related categories. Fifth, themes were reviewed and refined to ensure internal coherence and clear distinctions between individual and health system level barriers and facilitators, with explicit mapping to COM-B domains where appropriate. Sixth, themes were clearly defined and named, and detailed analytic summaries were developed, supported by illustrative participant quotations to maintain a strong link between interpretation and the original data. Regular analytic meetings with the wider study team were held to review emerging findings, interrogate assumptions, and strengthen interpretive depth.

## Results

### Participant characteristics

Table 1 summarizes participant demographics and sampling distribution by facility level and location. Data saturation was achieved after 22 interviews, which covered 8 AYPLHIV, 10 health workers, and 4 caregivers. Most participants (n = 12) were recruited from Health Centre IV, 5 from Health Centre III and 5 from hospitals. AYPLHIV, ages ranged from 12-24 years (median: 16.5). Five AYPLHIV were virally suppressed and three were non-suppressed after IAC. All caregivers were female, aged 39–65 years (median: 46.5). Health workers were aged 30 49 years (median: 40), with work experience that ranged 3–20 years

**Table 1:** Demographic profile of participants and sampling distribution across facilities.

No	Participant	Sex	Age	Education level	Facility level	Facility location	Duration on ART / Service
1	Adolescent (non-suppressed)	M	12	Primary	HC IV	Urban	12
2	Adolescent (suppressed)	F	14	Primary	HC III	Rural	13
3	Adolescent (non-suppressed)	M	15	Secondary	HC III	Rural	15
4	Adolescent (suppressed)	F	16	Secondary	HC IV	Rural	15
5	Adolescent (suppressed)	F	17	Primary	Hospital	Urban	1
6	Adolescent (suppressed)	M	19	Secondary	HC IV	Rural	2
7	Young adult (non-suppressed)	F	22	Secondary	Hospital	Rural	3
8	Young adult (suppressed)	M	24	Tertiary	HC IV	Urban	5
9	Caregiver	F	39	Primary	HC IV	Urban	14
10	Caregiver	F	40	Secondary	Hospital	Urban	17
11	Caregiver	F	53	Secondary	HC III	Rural	–
12	Caregiver	F	65	Tertiary	HC IV	Rural	–
13	Nurse	F	31	Certificate	HC III	Rural	4
14	Nurse	F	35	Diploma	HC IV	Urban	11
15	Clinical mentor	M	45	Degree	HC IV	Urban	18
16	Counsellor	F	30	Diploma	HC IV	Urban	3
17	Counsellor	M	37	Degree	HC IV	Rural	11
18	Counsellor	F	38	Diploma	HC IV	Rural	6
19	Counsellor	F	45	Degree	Hospital	Urban	15
20	ART Clinic In-charge	F	42	Diploma	HC IV	Rural	10
21	ART Clinic In-charge	M	49	Degree	Hospital	Urban	18
22	Facility In-charge	M	49	Degree	HC III	Rural	20

### Individual-level factors influencing VL suppression among AYPLHIV during IAC

Following the COM-B framework, we identified key perceived barriers and facilitators of ART adherence and IAC engagement from multiple perspectives of participants. While all three domains were examined, opportunity and motivation domains yielded richer data and are therefore more prominently represented in the findings.

### Capability factors influencing VL suppression among AYPLHIV during IAC

#### Theme: Forgetfulness and unintentional non-adherence.

Forgetfulness constituted a prominent psychological capability barrier to ART adherence and subsequent VL suppression. This barrier was most evident among school-going adolescents, where competing academic demands disrupted medication routines and clinic attendance. As one adolescent explained:

*“It’s not that I don’t want to go for counselling at the facility or take my drugs, I just forget sometimes, especially when there are so many activities at school, or I’m rushing for classes.”* (Adolescent, 14 years, suppressed, rural facility).

#### Theme: Establishment of routines.

Habitual medication-taking routines emerged as a key physical capability facilitator of adherence, directly supporting VL suppression among AYPLHIV receiving IAC. Participants described using structured reminders, particularly alarms, to reinforce consistent ART intake and clinic attendance. One client demonstrated:

*“As part of my adherence plan, I set up an alarm so that it reminds me of my clinic appointment and when to take my medication daily. This has helped me minimize forgetting”* (Young adult, 24 years, suppressed, urban facility).

#### Theme: Comprehension of ART and IAC.

Repeated counselling sessions enhanced psychological capability by clarifying IAC objectives. Many participants initially misunderstood the purpose of IAC but subsequently appreciated its role in supporting adherence and health outcomes. One young adult reflected:

*“Counselling made me realize it’s my responsibility to take my medicine and live well like anyone else. That’s why I suppressed after attending the counselling”* (Young adult, 24 years, suppressed, urban facility).

### Opportunity factors influencing VL suppression among AYPLHIV during IAC

#### Theme: School and work environment.

School and work settings acted as either facilitators or barriers to adherence, depending on the level of support and flexibility. AYPLHIV in supportive environments, where teachers or supervisors were aware of their HIV status, reported easier IAC attendance and consistent medication routines. One young adult noted:

*“Unlike my other sick friends who work in strict Indian-owned factories, I am a bit lucky; my boss is aware of my status, and he is lenient to me. It is easier for me to go to the clinic and come back on his permission whenever I want to”* (Young adult, 24 years suppressed, urban facility).

Conversely, rigid school or work schedules disrupted IAC attendance and delayed medication refills due to fear of reprimand. An adolescent described:

*“My school is far from the clinic, and the admin is tough, …remember the counselling is done on weekdays, which makes it hard for me to attend regularly without getting into trouble. That is why I didn’t go to the clinic for counselling or even to pick up drugs until I got holidays”* (Adolescent, 16 years suppressed, rural facility).

#### Theme: Economic hardship and caregiver support.

Economic hardship and limited caregiver support emerged as interconnected critical physical opportunity barriers to IAC engagement and ART adherence. Participants consistently reported poverty constraining access to food, transport, and basic necessities, leading to missed appointments and medication intake. One devastated non-supported adolescent said:

*“…You’re all on your own. You feel isolated, like no one really cares if you take the drugs or not. Sometimes I just give up on going to the clinic altogether. Most times, I have no money, no transport, no food, my clothes are dirty, and I have no soap. How can I go to a clinic with dirty clothes?”* (Adolescent, 15 years, non-suppressed, rural facility).

#### Theme: Stigma, discrimination and fear of disclosure.

Stigma, fear, and discrimination emerged as key interwoven social opportunity barriers that impeded engagement in IAC and ART adherence suppression. Participants attending particularly integrated clinics avoided unwanted disclosure. One married adolescent described fear concealing her medication from her partner:

*“I fear coz he may come to know my status. When he’s home, I hide the drugs, and I can’t move to the clinic for a refill because he will ask …you know, a man will run away from you if he comes to know.”* (Adolescent, 17 years, suppressed, urban facility).

#### Theme: Parental resentment.

Parental resentment emerged predominantly among perinatally infected adolescents who blamed their mothers for their HIV status, perceiving the condition as an undeserved burden. This diminished their motivation to regularly take medication. A 15-year-old orphaned non-suppressed participant reflected this sentiment:

*“…why would I take drugs daily? It was my mother’s fault. I am just suffering innocently… yet no one gives me a supportive hand”* (Adolescent, 15 years, non-suppressed, rural facility)

Health workers observed that unresolved resentment and limited reconciliation during counselling exacerbated non-adherence and poor outcomes. One nurse recounted one tragic case:

*“I’ve ever encountered many cases, there was one who blamed her mother until she died… She refused to take the medication despite IAC… The day she got to know that she was positive, and after 6 months of care, she was non-suppressed, she was blaming it on her mom. She got bedridden until she died.”* (Nurse, 11 years’ experience, rural facility).

#### Theme: Social class-based reluctance to engage with IAC services.

Health workers reported that adolescents from higher socioeconomic backgrounds often exhibited limited engagement with IAC, representing a major provider-identified barrier to VL suppression. AYPLHIV from wealthier families were noted to frequently resist follow-up home visits due to concerns about being associated with HIV services in their communities. As one counsellor explained:

*“Those from rich families don’t want to be monitored, followed up, or let anyone visit their home. They do not like to be linked to HIV services in the neighborhood. They prefer going for private care, which has no component of IAC”* (Counsellor, 11 years’ experience, urban facility).

Providers also observed that some adolescents from well-to-do families perceived IAC as redundant when private medical care or parental counselling was already available. A facility in charge noted:

*“We are challenged with kids from wealthy and educated families; some think we have nothing new to teach them. They have private doctors, or their parents do the counselling of the kids by themselves at home and see no point in attending the IAC sessions.”* (Facility In-charge, 20 years’ experience, rural facility).

### Motivation factors influencing VL suppression among AYPLHIV during IAC

Within the motivation domain, three primary contrasting themes emerged that illustrated how cognitive beliefs and emotional drivers jointly shaped AYPLHIV adherence and engagement with IAC that affect subsequent VL suppression.

#### Theme: Aspirational goals.

Aspirational goals strongly facilitated ART adherence and IAC engagement. Participants who articulated concrete ambitions, such as pursuing higher education or employment, reported adhering consistently to medication and attending IAC sessions to maintain health and achieve these objectives. One adolescent explained:

*“I want to be a nurse and help others. To do that, I must stay healthy by taking my medicine every day and keep the virus level low in my blood.”* (Adolescent, 16 years, suppressed, rural facility).

#### Theme: Faith and spiritual/religious beliefs.

Faith and religious ideologies exerted both facilitating and inhibiting effects on VL suppression among AYPLHIV during IAC. Participants reported that faith-based teachings emphasizing divine healing or prayer sometimes led to missed doses, non-attendance at IAC sessions, and persistent non-suppression. Conversely, religious leaders who reinforced adherence provided hope and motivation, supporting ART uptake and suppression. One adolescent noted:

*“… my pastor said ‘keep hopeful and have faith in God, take your medicine with faith as prescribed’... I know God will help me. I know I will not heal from the virus, but better health is all I need”.* (Adolescent, 17 years, suppressed, urban facility).

Others, however, experienced detrimental effects from teachings that prioritized spiritual deliverance over treatment. One participant confessed:

*“I was a victim of a born-again cult that told us to dump the drugs in the latrine, after being prayed for. That’s how I ended up not suppressing after IAC, honestly, I didn’t take the medicine”* (Young adult, 22 years, non-suppressed, rural facility).

#### Theme: Emotional distress and hopelessness.

Across transcripts, persistent emotional distress and hopelessness emerged as key psychological barriers to ART adherence. Participants consistently reported sadness, social isolation, and diminished motivation to adhere and attend IAC. One caregiver said;

*“There are days he just feels very low. He doesn’t want to talk to anyone, and he doesn’t even want to take the medicine or go for counselling. It feels heavy, like everything is too much for him.”* (Caregiver, 53 years old, not on ART, urban facility).

#### Theme: Substance abuse.

Substance use and abuse, particularly alcohol consumption, emerged as a significant barrier to VL suppression among AYPLHIV during IAC. Health workers and caregivers reported that adolescents who were frequently in environments where alcohol use was common often missed IAC appointments and exhibited poor adherence.

*“She/he tells you, that day I was high, I couldn’t turn up...and it happens like this for several of them with drinking behavior, they never come for counselling or for refills”* (Counsellor, 3 years’ experience, urban facility)

Similarly, one caregiver shared an experience:

*“I take care of two of my late brother’s sons, they go to the drinking joint, come back late, drunk, and fail to take their drugs... they don’t mind about coming for counselling even if you give them transport money”* (Caregiver, 65 years, not on ART, rural facility).

### Health system level barriers and facilitators of suppression among AYPLHIV during IAC

Health system factors operated as structural determinants alongside individual-level COM-B factors to influence VL suppression.

#### Theme: Inflexible IAC delivery schedule.

The rigid scheduling of IAC emerged as a prominent physical opportunity barrier, directly constraining VL suppression. Most school-going adolescents struggled to attend weekday sessions, while AYPLHIV, accustomed to longer ART refill intervals, found the monthly visits burdensome. One adolescent noted:

*“The counselling is only done during the week. How do I miss school to go see the counsellor on a weekday? How do I explain to my classmates and teacher that I had gone for counselling, that is why I missed” (Adolescent, 16 years, suppressed, rural facility).*

Similarly, young adults described the transition from three- or six-month drug refills to monthly IAC visits as disruptive: One young adult explained:

*“I don’t have time to over frequent the facility. I used to pick up my drugs after every three or six months; now these counselling sessions require me to come to the facility every month, which was not possible at all.” (Young adult, 24 years, suppressed, urban facility).*

#### Theme: Prolonged clinic waiting times.

Extended clinic waiting times emerged as key physical opportunity barriers to IAC engagement and VL suppression among AYPLHIV. Almost all participants reported that service delays often lead to missed or postponed visits. A clinical mentor shared his experience and explained:

*“Due to long queues, sometimes they just say, ‘I’ll come back tomorrow,’ but they don’t, and then they repeat the same next time they find a long line.”* (Clinical mentor, 13 years’ experience, urban facility).

Similarly, a school-going adolescent voiced frustration with the long lines:

*“You know the clinic runs on weekdays, sometimes I escape from school during lunch time, I reach the facility, and the line is long… I end up escaping from the clinic again without being attended to so that I catch up with class time”* (Adolescent, 14 years, suppressed, rural facility).

#### Theme: Limited clinic responsiveness to AYPLHIV needs.

All Counsellors highlighted that the lack of tailored follow-up, including home visits or individualized support, left key adherence barriers unaddressed during IAC. One counselor explained:

*“During IAC, they (adolescents) come up with issues such as a lack of food or transport means. There is nothing much we can do apart from giving him/her counselling information and the drugs, because that’s all we can afford.”* (Counsellor, 11 years’ experience, rural facility)*.*

Another provider noted:

*“We are not providing much and following up much. Most of the time we use phone calls, but this category lacks phones or stays in remote areas without network... we can’t physically reach them due to lack of transport”* (ART clinic in-charge, 10 years’ experience, rural facility).

#### Theme: Community health worker and peer support programs.

Community-level support through community health workers, expert clients and peers was reported in facilities that had these programs as a strong facilitator. Active VHT and peer involvement through home visits were described as enablers for reducing missed doses during IAC. A clinical mentors stated:

*“We started peer groups for the unsuppressed. They meet, share experiences, and one difficult-to-counsel girl and many others started taking her drugs regularly after hearing others’ stories.”* (Clinical mentor, 13 years’ experience, urban facility).

A supported adolescent explained:

*“The musawo from the village (VHT) visits and teaches us about the importance of taking medicine on time…They count my medicine to see if I missed.”* (Adolescent, 14 years, rural facility).

#### Theme: Social media use (WhatsApp groups).

Social media-based peer support groups, particularly WhatsApp groups, emerged as a key system-level facilitator of VL suppression among AYPLHIV enrolled in IAC. Providers reported that these platforms enabled continuous counselling engagement, peer-to-peer encouragement, and normalization of the treatment experience beyond the clinic setting. Adolescents who had exposure to social media groups reported getting inspired. One adolescent shared:

*“Seeing others managing this thing (referring to ART) and living happily with HIV in our WhatsApp group gave me hope that I can also have a good life.”* (Young adult, 22 years, non-suppressed, rural facility).

### Summary of findings

Overall, the findings demonstrate that viral load suppression among AYPLHIV during IAC is shaped by interacting individual and health system factors. Within the COM-B framework, barriers included forgetfulness, economic hardship, stigma and non-disclosure, parental resentment, emotional distress, and substance abuse, while facilitators comprised reminder routines, improved understanding of ART and IAC, caregiver support, future aspirations, and supportive religious beliefs. At the health system level, inflexible IAC schedules, prolonged clinic waiting times, and limited responsiveness to adolescents’ needs constrained engagement, whereas peer support, community health worker involvement, and WhatsApp-based support strengthened adherence and participation in IAC.

## Discussion

This study explored individual and health system-level factors that affected ART adherence and engagement of AYPLHIV in IAC as pathway to VL suppression in east central Uganda. Using the COM-B framework, we identified key physical and psychological capability-related barriers, including forgetfulness, unintentional non-adherence, and limited understanding of ART and IAC that compromised adherence and virological outcomes. These findings align with prior evidence linking low health literacy and misunderstanding of ART to suboptimal adherence and VL suppression [14,15,22]. Conversely, routine-support strategies such as mobile phone alarms and reminders facilitated habitual medication use, thereby reinforcing individual capability and improving the probability of VL suppression following IAC [23]. These findings emphasize persistent gaps in HIV-related health literacy while highlighting the potential for integrating low-cost, scalable reminder systems and youth-tailored education into IAC programs to demystify ART and strengthen sustained adherence.

Opportunity-related barriers to ART adherence and IAC engagement in this study comprised economic hardship, inflexible school and work schedules, and limited caregiver involvement, alongside stigma, fear of discrimination, and non-disclosure. Parental resentment and social class-related service aversion are unique findings that were largely provider-reported. AYPLHIV from higher socioeconomic strata avoided public HIV services due to reputational concerns, often resisting follow-up or home-based care and preferring to seek private services that do not provide IAC, thereby interrupting adherence support. These barriers are known to reduce social support, constrain access to adherence-enabling environments, and lead to intentional disengagement from structured counselling [24–26]. Parental resentment and social class-related service aversion are unique findings that were largely provider-reported and warrant further empirical validation, as they remain underexplored in Ugandan settings. Nonetheless, these findings align with prior evidence linking stigma, disclosure challenges, and structural constraints to suboptimal ART adherence and VL suppression [23,27–31]. Conversely, supportive caregivers and accommodating school or workplace environments facilitated IAC engagement and adherence, consistent with existing literature elsewhere [24,28,32]. Overall, the findings in this domain suggest that achieving optimal adherence extends beyond counselling, calling for the integration of IAC with interventions that strengthen enabling social and structural environments for consistent medication-taking among AYPLHIV.

Within the motivation domain, adherence and IAC engagement were primarily driven by emotional distress characterized sadness, social isolation, and diminished motivation to adhere. This pattern corroborates evidence identifying emotional distress as a key determinant of poor adherence among adolescents, particularly males [23,27)]. Conversely, aspirational goals and spiritual/religious beliefs functioned as key facilitators of VL suppression. Future-oriented aspirations and internalized values are known to strengthen adherence by enhancing self-efficacy and resilience. Religious beliefs exert a bidirectional effect where some undermine adherence through preference for spiritual healing over biomedical care, while others reinforced ART uptake via faith-based encouragement and social support [27,33–35]. These findings indicate that integrating goal-oriented and faith-sensitive counselling with structured mental health support within IAC may reduce substance abuse, mitigate emotional distress, and improve VL suppression among AYPLHIV.

At the health-system level, predominant barriers to VL suppression included inflexible IAC scheduling that conflicted with school attendance, prolonged clinic waiting times, and limited responsiveness to adolescent-specific needs. These barriers reflect persistent structural limitations in resource-constrained Ugandan settings, such as understaffing, long distances, and inadequate adolescent-responsive services. These constraints reduce service accessibility and continuity of engagement, thereby disrupting adherence behaviors and limiting the effectiveness of IAC [26,32,36,37]. Conversely, facilitators included structured community peer support programs and digital peer platforms (WhatsApp groups) that enhance social connectedness. Digital platforms extended support beyond clinic encounters by providing adherence prompts and psychosocial reinforcement. These findings are consistent with evidence that peer engagement and acceptable digital platforms enhance retention in care [16,38]. Aligning IAC delivery with flexible, adolescent-responsive models that integrate peer and digital support is critical to optimizing engagement and improving VL suppression outcomes.

This study had several strengths, including the use of the COM-B framework to provide a systematic, theory-informed understanding of barriers and facilitators to viral load suppression, and the inclusion of diverse participant perspectives that enhanced the depth and credibility of the findings. However, several limitations should be considered. Data were collected after completion of IAC and receipt of viral load results, which may have introduced recall bias. In addition, discussions on ART adherence and substance use may have been influenced by social desirability bias, with participants potentially underreporting behaviors perceived as undesirable. Although maximum variation sampling enhanced the richness of the data, the qualitative design and context-specific nature of the study may limit the transferability of the findings beyond East-Central Uganda. Furthermore, some facilitators, such as WhatsApp-based peer support, were age- and technology-dependent and may not apply to younger adolescents or settings with limited digital access. Lastly, excluding people who were clinically unstable or had advanced HIV disease may have removed perspectives of people with very important barriers and the poorest outcomes.

## Conclusion

Virally non-suppressed adolescents and young people living with HIV in the Busoga sub-region face multiple interacting individual and health-system barriers that undermine ART adherence and participation in IAC that compromise achievement of VL suppression. Economic hardship, stigma, emotional distress, restrictive school environments, long waiting times, and negative provider interactions adversely affected engagement in care and adherence. Although future aspirations, spirituality, and caregiver or peer support enhanced motivation, these factors were often insufficient to overcome structural barriers. These findings suggest that facility-based counselling alone is inadequate to optimize IAC outcomes. Improving VL suppression through AIC will require multicomponent, youth-responsive IAC delivery strategies that integrate peer support, SMS-based adherence reminders, community-based differentiated service delivery models, flexible clinic schedules, and reduced waiting times. Before widespread implementation, these strategies should be evaluated through rigorous implementation research to determine their feasibility, acceptability, effectiveness, fidelity, and scalability within routine HIV care in Uganda.
