# Supplementary material for: Barriers and facilitators to viral load suppression among adolescents and young people living with HIV receiving intensive adherence counselling in east central Uganda: A COM-B model-informed qualitative study
Source: PLOS Glob Public Health. 2026 Aug 20;6(8):e0007131. doi: 10.1371/journal.pgph.0007131 (PMC13492741; doi:10.1371/journal.pgph.0007131)
Supplement: Supplementary file 1 [file pgph.0007131.s003.pdf]

Point by point response matrix to reviewer comments

We sincerely thank the Editor and both reviewers for their careful assessment of our manuscript and their constructive comments. We have addressed all comments in the revised manuscript. Our responses are presented below, with reviewer comments reproduced in column 1, followed by our responses in column 2. All modifications have been incorporated into the revised manuscript, and the corresponding line numbers are indicated.

Reviewer #1:

**comment**	**Response**
This is a well-written qualitative study addressing an important public health issue among adolescents and young people living with HIV (AYPLHIV). The use of the COM-B framework provides a clear theoretical basis for exploring barriers and facilitators to viral load suppression during intensive adherence counselling (IAC). The findings are relevant and have practical implications. The following revisions would strengthen the manuscript.	Thank you for this encouraging comment.
1. Correct the inconsistent capitalisation of "Substance abuse" in the Results section.	Revised accordingly.<br><br>Line 46
2. Ensure consistent use of the terms viral load suppression, viral suppression, and VL suppression throughout the manuscript.	The suggested revision has been incorporated throughout the manuscript.
3. Review the manuscript for minor grammatical and formatting inconsistencies.	The suggested revisions have been incorporated throughout the manuscript.
**Abstract**	
4. State the qualitative design explicitly (e.g., descriptive qualitative study).	Revised accordingly.

	Line 35<br><br>Study design stated "A descriptive exploratory qualitative study design "
5. Clarify that health system factors emerged inductively and complemented the COM-B framework.	Revised accordingly.<br><br>Line 37-38
6. Briefly indicate the practical implications of the findings.	We agree with the reviewer and have modified the text to improve clarity<br><br>Line 50-53
**Introduction**	
7. The background is comprehensive and clearly establishes the research gap.	Thank you for this acknowledgement
8. Consider shortening the description of IAC to improve readability.	Revised accordingly.<br><br>Line 69-75
**Methods**	
9. Briefly justify why the COM-B model was selected over other behavioural frameworks. Methods	Thank you for this helpful suggestion. We have revised the manuscript accordingly<br><br>Line 98-101,
10. Clarify the study period. The manuscript states interviews were conducted between 20 September 2025 and 15 January 2025, which appears to be a typographical error.	Thank you for this helpful suggestion. We have revised the manuscript accordingly.<br><br>Line 132: the typographical error has been corrected
11. Explain how the researchers determined that thematic saturation had been reached across the three participant groups.	This explanation has been included.<br><br>Line 123-127,
12. Specify whether participant quotations were	We agree with the reviewer and have modified the text to improve clarity

edited for readability after translation.	Some of the participant quotations were edited to improve readability after translation
**Results**	
13. The themes are clearly organized according to the COM-B framework.	Thank you for this comment
14. Consider reducing repetition where similar barriers (e.g., stigma and non-disclosure) appear under multiple themes.	Thanks for this observation, these repetitions have been removed
15. Include a brief summary paragraph at the end of the Results highlighting the most important findings before moving to the discussion	Line 432-440 This paragraph has been added
**Discussion.**	
16. The discussion is well linked to previous literature	Thank you for acknowledging this.
17. Expand on the novel findings regarding parental resentment and social class-related reluctance and discuss their implications for future interventions.<br><br>18. Distinguish more clearly between findings directly supported by participants and those primarily reported by health workers.	Thank you for this helpful suggestion. We have revised the manuscript accordingly<br><br>Line 461-469, additional elaboration of these findings has been added.<br><br>The distinction between what participants reported and that which health workers reported are clearly distinguished in the results themes
**Limitations**<br><br>19. In addition to recall bias, acknowledge the possibility of social desirability bias, particularly when discussing ART adherence and substance use.	Revised accordingly.<br><br>Line 487-499
20. Mention that the findings may not be transferable beyond East Central Uganda	Revised accordingly.

because of the qualitative design.<br>Conclusion	Line 487-499
21. The conclusions are generally supported by the findings.	
22. Consider emphasizing that the recommended interventions should be evaluated through implementation research before widespread adoption.	Revised accordingly.<br><br>Line 521-526,

Reviewer 2

Reviewer #2:

**comment**	**Response**
Line 30 – 31. This study examined the facilitators and barriers related to ART adherence post IAC. However, you extend adherence to viral load suppression, which is a biological outcome. This stretch from adherence to VL suppression is not supported by the methodology used in this study. “Adherence to ART” is not equivalent to “VL suppression”. This should therefore be revised. The framework (COM-B) in this study is related to behavioral concepts. However, viral load suppression is not a behavioral concept. Please change this throughout the manuscript. VL seems to have replaced “adherence to ART” in many sections of the manuscript (eg. Line 89-90). Since this is main concept being examined in the study, please be consistent in what was being examined in the study and revise language that equates adherence to VL suppression.	This is by far the most excellent and logical insight to improve the alignment of this study. Thank you very much.<br><br>The suggested revision has been incorporated throughout the manuscript.

Line 32 – Sentence missing some conjunctions. Should read “was conducted among Participants”	Revised accordingly.
Line 49 – Your abstract conclusion is possible but is overstretching. Since adherence is the main behavioral component being examined, you cinclusion should reflect this and not VL suppression.	The conclusion has been focused on adherence and IAC engagement, which are pathways to Viral load suppression
Line 55- 66. The comparisons use different age groups: AYPLHIV aged 10–24, adolescents aged 15–19, and “children.” Please use age-comparable estimates.	The error in the sentence has been corrected. The estimate provided is an overall estimate.
Line 96-97. The COM-B target behaviour is not clearly defined. Is the behaviour daily ART taking, attending IAC, IAC engagement, attending clinic or obtaining refills? Please define the primary target behaviour and distinguish it from the clinical outcome of viral suppression.	We agree with the reviewer and have modified the text to improve clarity<br><br>This has been highlighted; the primary target behaviour was ART adherence during IAC, while the secondary behavior was continuous engagement in IAC.
Line 122- Excluding people who were clinically unstable or had advanced HIV disease may remove perspectives of people with very important barriers and the poorest outcomes. Please acknowledge this limitation.	We agree with the reviewer and have modified the text to improve clarity<br><br>This limitation has been acknowledged in the limitations section below the discussion section.
Line 145- prolonged engagement and interviews do not necessarily improve credibility of the interviews. I suggest deleting this or revising it.	This statement has been deleted.
Line 150 – Multidisciplinary team does not improve reflexivity directly. Please revise this.	This statement has been deleted.

Line 209 - Forgetfulness was described as a Physical capability barrier to ART adherence. Is this consistent with the Com-B. Forgetfulness fits better into psychological component.	This anomaly has been corrected; forgetfulness is a psychological capability factor rather than physical, as stated before.
Line 229 – Delete one of the sentences about a young adult reflecting and an additional sentence that says a young adult also explained.	The repetition has been deleted.
Line 441- Izudi et al not consistent with the referencing in the rest of the manuscript.<br>Line 463 – Please revise "these factors likely diminish motivation to compromise consistent ART" for clarity.	The referencing style has been aligned
Strengths<br>The study addresses an important public health topic in a marginalized and at-risk population.<br>The study guided by a clear conceptual framework giving it theoretical structure.	Thank you for acknowledging the importance of this paper
