# Supplementary material for: Barriers and facilitators to viral load suppression among adolescents and young people living with HIV receiving intensive adherence counselling in east central Uganda: A COM-B model-informed qualitative study
Source: PLOS Glob Public Health. 2026 Aug 20;6(8):e0007131. doi: 10.1371/journal.pgph.0007131 (PMC13492741; doi:10.1371/journal.pgph.0007131)
Supplement: Supplementary file 2 [file pgph.0007131.s001.docx]

**Peer review comments**

**Line 30 – 31**. This study examined the facilitators and barriers related to ART adherence post IAC. However, you extend adherence to viral load suppression which is a biological outcome. This stretch from adherence to VL suppression is not supported by the methodology used in this study. “Adherence to ART” is not equivalent to “VL suppression”. This should therefore be revised. The framework (COM-B) in this study is related to behavioral concepts. However, viral load suppression is not a behavioral concept. Please change this throughout the manuscript. VL seems to have replaced “adherence to ART” in many sections of the manuscript (eg. Line 89-90). Since this is main concept being examined in the study, please be consistent in what was being examined in the study and revise language that equates adherence to VL suppression.

**Line 32** – Sentence missing some conjunctions. Should read “was conducted among

Participants”

**Line 49 –** Your abstract conclusion is possible but is overstretching. Since adherence is the main behavioral component being examined, you cinclusion should reflect this and not VL suppression.

**Line 55- 66.** The comparisons use different age groups: AYPLHIV aged 10–24, adolescents aged 15–19, and “children.” Please use age-comparable estimates.

**Line 96-97.** The COM-B target behaviour is not clearly defined. Is the behaviour daily ART taking, attending IAC, IAC engagement, attending clinic or obtaining refills? Please define the primary target behaviour and distinguish it from the clinical outcome of viral suppression.

**Line 122**- Excluding people who were clinically unstable or had advanced HIV disease may remove perspectives of people with very important barriers and the poorest outcomes. Please acknowledge this limitation.

**Line 145-** prolonged engagement and interviews do not necessarily improve credibility of the interviews. I suggest deleting this or revising it.

Line 150 – Multidisciplinary team does not improve reflexivity directly. Please revise this.

Line 209 - Forgetfulness was described as a Physical capability barrier to ART adherence. Is this consistent with the Com-B. Forgetfulness fits better into psychological component.

**Line 229 –** Delete one of the sentences about a young adult reflecting and an additional sentence that says a young adult also explained.

**Line 441**- Izudi et al not consistent with the referencing in the rest of the manuscript.

**Line 463** – Please revise “these factors likely diminish motivation to compromise consistent ART” for clarity.

**Strengths**

The study addresses an important public health topic in a marginalized and at-risk population.

The study guided by a clear conceptual framework giving it theoretical structure.
