# Supplementary material for: Barriers and facilitators to viral load suppression among adolescents and young people living with HIV receiving intensive adherence counselling in east central Uganda: A COM-B model-informed qualitative study
Source: PLOS Glob Public Health. 2026 Aug 20;6(8):e0007131. doi: 10.1371/journal.pgph.0007131 (PMC13492741; doi:10.1371/journal.pgph.0007131)
Supplement: Peer review history

# Peer review: Decision Letter 0

**DOI:** 10.1371/journal.pgph.0007131.r001

**Authors:** Sanghyuk Shin

21 Jul 2026

PGPH-D-26-02087

Barriers and facilitators to viral load suppression among adolescents and young people living with HIV receiving intensive adherence counselling in east central Uganda: A COM-B model informed qualitative study.

PLOS Global Public Health

Dear Dr. Ejalu,

Thank you for submitting your manuscript to PLOS Global Public Health. After careful consideration, we feel that it has merit but does not fully meet PLOS Global Public Health’s publication criteria as it currently stands. Therefore, we invite you to submit a revised version of the manuscript that addresses the points raised during the review process.

Please submit your revised manuscript by Aug 20 2026 11:59PM. If you will need more time than this to complete your revisions, please reply to this message or contact the journal office at globalpubhealth@plos.org. When you're ready to submit your revision, log on to https://www.editorialmanager.com/pgph/ and select the 'Submissions Needing Revision' folder to locate your manuscript file.

Please include the following items when submitting your revised manuscript:

- A letter that responds to each point raised by the editor and reviewer(s). You should upload this letter as a separate file labeled 'Response to Reviewers'.
- A marked-up copy of your manuscript that highlights changes made to the original version. You should upload this as a separate file labeled 'Revised Manuscript with Track Changes'.
- An unmarked version of your revised paper without tracked changes. You should upload this as a separate file labeled 'Manuscript'.

Guidelines for resubmitting your figure files are available below the reviewer comments at the end of this letter.

As the corresponding author, your ORCID iD is verified in the submission system and will appear in the published article. PLOS supports the use of ORCID, and we encourage all coauthors to register for an ORCID iD and use it as well. Please encourage your coauthors to verify their ORCID iD within the submission system before final acceptance, as unverified ORCID iDs will not appear in the published article. *Only*  the individual author can complete the verification step; PLOS staff *cannot*  verify ORCID iDs on behalf of authors.

We look forward to receiving your revised manuscript.

Kind regards,

Sanghyuk S Shin

Academic Editor

PLOS Global Public Health

Journal Requirements:

1. Please ensure that your Ethics Statement is available in its entirety at the beginning of your Methods section, under a subheading 'Ethics Statement'.

2. Please amend your detailed Financial Disclosure statement. This is published with the article. It must therefore be completed in full sentences and contain the exact wording you wish to be published.

a. State the initials, alongside each funding source, of each author to receive each grant.

b. State what role the funders took in the study. If the funders had no role in your study, please state: “The funders had no role in study design, data collection and analysis, decision to publish, or preparation of the manuscript.”

c. If any authors received a salary from any of your funders, please state which authors and which funders.

3. Your manuscript is missing the following sections: Introduction. Please ensure these are present, and in the correct order, and that any references to subheadings in your main text are correct. An outline of the required sections can be consulted in our submission guidelines here: [LINK]

https://journals.plos.org/globalpublichealth/s/submission-guidelines#loc-parts-of-a-submission

4. We have noticed that you have uploaded Supporting Information files, but you have not included a list of legends. Please add a full list of legends for your Supporting Information files after the references list.

5. In the online submission form, you indicated that "The datasets used and/or analyzed during the current study are available from the corresponding author on request.".

All PLOS journals now require all data underlying the findings described in their manuscript to be freely available to other researchers, either

1. In a public repository,

2. Within the manuscript itself, or

3. Uploaded as supplementary information.

This policy applies to all data except where public deposition would breach compliance with the protocol approved by your research ethics board. If your data cannot be made publicly available for ethical or legal reasons (e.g., public availability would compromise patient privacy), please explain your reasons by return email and your exemption request will be escalated to the editor for approval. Your exemption request will be handled independently and will not hold up the peer review process, but will need to be resolved should your manuscript be accepted for publication. One of the Editorial team will then be in touch if there are any issues.

If the reviewer comments include a recommendation to cite specific previously published works, please review and evaluate these publications to determine whether they are relevant and should be cited. There is no requirement to cite these works unless the editor has indicated otherwise.

Please review your reference list to ensure that it is complete and correct. If you have cited papers that have been retracted, please include the rationale for doing so in the manuscript text, or remove these references and replace them with relevant current references. Any changes to the reference list should be mentioned in the rebuttal letter that accompanies your revised manuscript. If you need to cite a retracted article, indicate the article’s retracted status in the References list and also include a citation and full reference for the retraction notice.

Additional Editor Comments (if provided):

[Note: HTML markup is below. Please do not edit.]

Reviewers' comments:

Reviewer's Responses to Questions

**Comments to the Author**

1. Does this manuscript meet PLOS Global Public Health’s publication criteria? Is the manuscript technically sound, and do the data support the conclusions? The manuscript must describe methodologically and ethically rigorous research with conclusions that are appropriately drawn based on the data presented.

Reviewer #1: Yes

Reviewer #2: Yes

**********

2. Has the statistical analysis been performed appropriately and rigorously?

Reviewer #1: N/A

Reviewer #2: N/A

**********

3. Have the authors made all data underlying the findings in their manuscript fully available (please refer to the Data Availability Statement at the start of the manuscript PDF file)?

The PLOS Data policy requires authors to make all data underlying the findings described in their manuscript fully available without restriction, with rare exception. The data should be provided as part of the manuscript or its supporting information, or deposited to a public repository. For example, in addition to summary statistics, the data points behind means, medians and variance measures should be available. If there are restrictions on publicly sharing data—e.g. participant privacy or use of data from a third party—those must be specified.

Reviewer #1: No

Reviewer #2: Yes

**********

4. Is the manuscript presented in an intelligible fashion and written in standard English?

PLOS Global Public Health does not copyedit accepted manuscripts, so the language in submitted articles must be clear, correct, and unambiguous. Any typographical or grammatical errors should be corrected at revision, so please note any specific errors here.

Reviewer #1: Yes

Reviewer #2: Yes

**********

5. Review Comments to the Author

Please use the space provided to explain your answers to the questions above. You may also include additional comments for the author, including concerns about dual publication, research ethics, or publication ethics. (Please upload your review as an attachment if it exceeds 20,000 characters)

Reviewer #1: This is a well-written qualitative study addressing an important public health issue among adolescents and young people living with HIV (AYPLHIV). The use of the COM-B framework provides a clear theoretical basis for exploring barriers and facilitators to viral load suppression during intensive adherence counselling (IAC). The findings are relevant and have practical implications. The following revisions would strengthen the manuscript.

General Comments

1. Correct the inconsistent capitalization of "Substance abuse" in the Results section.

2. Ensure consistent use of the terms viral load suppression, viral suppression, and VL suppression throughout the manuscript.

3. Review the manuscript for minor grammatical and formatting inconsistencies.

Abstract

4. State the qualitative design explicitly (e.g., descriptive qualitative study).

5. Clarify that health system factors emerged inductively and complemented the COM-B framework.

6. Briefly indicate the practical implications of the findings.

Introduction

7. The background is comprehensive and clearly establishes the research gap.

8. Consider shortening the description of IAC to improve readability.

9. Briefly justify why the COM-B model was selected over other behavioural frameworks.

Methods

10. Clarify the study period. The manuscript states interviews were conducted between 20 September 2025 and 15 January 2025, which appears to be a typographical error.

11. Explain how the researchers determined that thematic saturation had been reached across the three participant groups.

12. Specify whether participant quotations were edited for readability after translation.

Results

13. The themes are clearly organized according to the COM-B framework.

14. Consider reducing repetition where similar barriers (e.g., stigma and non-disclosure) appear under multiple themes.

15. Include a brief summary paragraph at the end of the Results highlighting the most important findings before moving to the Discussion.

Discussion

16. The discussion is well linked to previous literature.

17. Expand on the novel findings regarding parental resentment and social class-related reluctance and discuss their implications for future interventions.

18. Distinguish more clearly between findings directly supported by participants and those primarily reported by health workers.

Limitations

19. In addition to recall bias, acknowledge the possibility of social desirability bias, particularly when discussing ART adherence and substance use.

20. Mention that the findings may not be transferable beyond East Central Uganda because of the qualitative design.

Conclusion

21. The conclusions are generally supported by the findings.

22. Consider emphasizing that the recommended interventions should be evaluated through implementation research before widespread adoption.

Reviewer #2: Line 30 – 31. This study examined the facilitators and barriers related to ART adherence post IAC. However, you extend adherence to viral load suppression which is a biological outcome. This stretch from adherence to VL suppression is not supported by the methodology used in this study. “Adherence to ART” is not equivalent to “VL suppression”. This should therefore be revised. The framework (COM-B) in this study is related to behavioral concepts. However, viral load suppression is not a behavioral concept. Please change this throughout the manuscript. VL seems to have replaced “adherence to ART” in many sections of the manuscript (eg. Line 89-90). Since this is main concept being examined in the study, please be consistent in what was being examined in the study and revise language that equates adherence to VL suppression.

Line 32 – Sentence missing some conjunctions. Should read “was conducted among

Participants”

Line 49 – Your abstract conclusion is possible but is overstretching. Since adherence is the main behavioral component being examined, you cinclusion should reflect this and not VL suppression.

Line 55- 66. The comparisons use different age groups: AYPLHIV aged 10–24, adolescents aged 15–19, and “children.” Please use age-comparable estimates.

Line 96-97. The COM-B target behaviour is not clearly defined. Is the behaviour daily ART taking, attending IAC, IAC engagement, attending clinic or obtaining refills? Please define the primary target behaviour and distinguish it from the clinical outcome of viral suppression.

Line 122- Excluding people who were clinically unstable or had advanced HIV disease may remove perspectives of people with very important barriers and the poorest outcomes. Please acknowledge this limitation.

Line 145- prolonged engagement and interviews do not necessarily improve credibility of the interviews. I suggest deleting this or revising it.

Line 150 – Multidisciplinary team does not improve reflexivity directly. Please revise this.

Line 209 - Forgetfulness was described as a Physical capability barrier to ART adherence. Is this consistent with the Com-B. Forgetfulness fits better into psychological component.

Line 229 – Delete one of the sentences about a young adult reflecting and an additional sentence that says a young adult also explained.

Line 441- Izudi et al not consistent with the referencing in the rest of the manuscript.

Line 463 – Please revise “these factors likely diminish motivation to compromise consistent ART” for clarity.

Strengths

The study addresses an important public health topic in a marginalized and at-risk population.

The study guided by a clear conceptual framework giving it theoretical structure.

**********

6. PLOS authors have the option to publish the peer review history of their article (what does this mean?). If published, this will include your full peer review and any attached files.

**Do you want your identity to be public for this peer review?**  If you choose “no”, your identity will remain anonymous but your review may still be made public.

For information about this choice, including consent withdrawal, please see our Privacy Policy.

Reviewer #1: **Yes:** Kevin Ouma OJIAMBO

Reviewer #2: No

**********

[NOTE: If reviewer comments were submitted as an attachment file, they will be attached to this email and accessible via the submission site. Please log into your account, locate the manuscript record, and check for the action link "View Attachments". If this link does not appear, there are no attachment files.]

Figure Resubmissions:

While revising your submission, we strongly recommend that you use PLOS’s NAAS tool (https://ngplosjournals.pagemajik.ai/artanalysis) to test your figure files. NAAS can convert your figure files to the TIFF file type and meet basic requirements (such as print size, resolution), or provide you with a report on issues that do not meet our requirements and that NAAS cannot fix.

After uploading your figures to PLOS’s NAAS tool - https://ngplosjournals.pagemajik.ai/artanalysis, NAAS will process the files provided and display the results in the "Uploaded Files" section of the page as the processing is complete. If the uploaded figures meet our requirements (or NAAS is able to fix the files to meet our requirements), the figure will be marked as "fixed" above. If NAAS is unable to fix the files, a red "failed" label will appear above. When NAAS has confirmed that the figure files meet our requirements, please download the file via the download option, and include these NAAS processed figure files when submitting your revised manuscript.

Attachment Submitted filename: Peer review comments.docx

# Author comment: Author response to Decision Letter 1

**DOI:** 10.1371/journal.pgph.0007131.r002

26 Jul 2026

Attachment Submitted filename: Response to reviewers v1.pdf

# Editor report: Decision Letter 1

**DOI:** 10.1371/journal.pgph.0007131.r003

**Authors:** Sanghyuk Shin

31 Jul 2026

Barriers and facilitators to viral load suppression among adolescents and young people living with HIV receiving intensive adherence counselling in east central Uganda: A COM-B model informed qualitative study.

PGPH-D-26-02087R1

Dear Mr Ejalu,

We are pleased to inform you that your manuscript 'Barriers and facilitators to viral load suppression among adolescents and young people living with HIV receiving intensive adherence counselling in east central Uganda: A COM-B model informed qualitative study.' has been provisionally accepted for publication in PLOS Global Public Health.

Before your manuscript can be formally accepted you will need to complete some formatting changes, which you will receive in a follow up email. A member of our team will be in touch with a set of requests.

Please note that your manuscript will not be scheduled for publication until you have made the required changes, so a swift response is appreciated.

IMPORTANT: The editorial review process is now complete. PLOS will only permit corrections to spelling, formatting or significant scientific errors from this point onwards. Requests for major changes, or any which affect the scientific understanding of your work, will cause delays to the publication date of your manuscript.

If your institution or institutions have a press office, please notify them about your upcoming paper to help maximize its impact. If they'll be preparing press materials, please inform our press team as soon as possible -- no later than 48 hours after receiving the formal acceptance. Your manuscript will remain under strict press embargo until 2 pm Eastern Time on the date of publication. For more information, please contact globalpubhealth@plos.org.

Thank you again for supporting Open Access publishing; we are looking forward to publishing your work in PLOS Global Public Health.

Best regards,

Sanghyuk S Shin

Academic Editor

PLOS Global Public Health

***********************************************************

Reviewer Comments (if any, and for reference):
